# Case Report: Molecular Detection of *Dirofilaria repens* in an Italian Patient after a Stay in Tanzania

**DOI:** 10.4269/ajtmh.20-1360

**Published:** 2021-05-03

**Authors:** Donato Antonio Raele, Nicola Pugliese, Gianfranco La Bella, Agata Calvario, Maria Scarasciulli, Ilaria Vasco, Giovanna La Salandra, Maria Assunta Cafiero

**Affiliations:** 1 Istituto Zooprofilattico Sperimentale della Puglia e della Basilicata, Foggia, Italy;; 2 Dipartimento d Medicina Veterinaria, Università degli Studi di Bari, Valenzano, Italy;; 3 Laboratorio di Virologia U.O.C. Microbiologia e Virologia AOU Policlinico, Bari, Italy;; 4 Laboratorio di Virologia U.O.C. Microbiologia e Virologia, Dipartimento Interdisciplinare di Medicina, Università degli Studi di Bari, Valenzano, Italy

## Abstract

A 35-year-old man was admitted to a hospital in the south of Italy because of a periocular nodule and subpalpebral edema. The patient reported having been stayed in Tanzania five months before. Hematologic parameters were within the normality range, the *Acanthocheilonema viteae* ELISA did not detect significant levels of antifilarial IgG, and no further symptoms were described. The surgical inspection of the nodule led to the isolation of two filarioid parasites, identified as *Dirofilaria repens* by scanning electron microscope (SEM), and then by molecular assays. Knott’s test did not reveal microfilaremia, whereas loop-mediated isothermal amplification and PCR detected *D. repens* DNA. The patient was treated with doxycycline, and he was found no more positive at the follow-up.

## INTRODUCTION

Human dirofilariasis by *Dirofilaria* (*Nochtiella*) *repens* (Nematoda, Onchocercidae) is a mosquito-borne parasitic zoonosis, which mostly affects dogs in the Old World.^1,2^ However, the disease is becoming a matter of growing concern because several factors, such as the frequent lack of clinical signs in both humans and dogs, the limited range of treatments, and the climate changes, are contributing to its wide circulation in Europe.^3^ In fact, an increasing number of reports are being recorded not only from Italy and other European countries bordering the Mediterranean Sea, where dirofilariasis is considered endemic,^2,4^ but also from Eastern Europe, in particular Russia and former Soviet countries,^5^ Northern Africa, and Middle and Far East.^3^

Microfilaremic domestic dogs are considered the most important *Dirofilaria repens* reservoir, despite wild dogs and cats being involved, too.^4,6^ Microfilariae produced by female worms may invade the bloodstream of the natural reservoir of the parasite and be ingested by mosquitoes, where they molt and develop into the infesting stage (larva L3), which, in turn, can be released or transmitted by bite to other hosts.^7^

In humans, dirofilariasis is often asymptomatic, and the parasite usually does not develop into the adult form,^4^ but larvae may spread through the bloodstream up to reach the subcutaneous tissues, often forming nodules.^8^

The golden standard for the diagnosis of canine or feline dirofilariasis is the Knott’s test and its variants,^9,10^ based on the microscopic detection and morphological identification of microfilariae from blood. Considering that expert operators are needed to correctly recognize and identify microfilariae, molecular tests have been developed to selectively amplify by PCR^11^ and loop-mediated isothermal amplification (LAMP)^12^ species-specific regions of the *D. repens* genome. However, the intermittence of microfilaremia, along with the low concentration in blood, makes challenging the prompt diagnosis of dirofilariasis by *D. repens* in dogs.^7^ The issue is even more critical for humans because the microfilarial stage is absent or, at least, very rare.^13^ In those cases, diagnosis may be achieved by combining anamnestic data (especially those about travels or lifestyle) and the clinical picture, with ultrasonography^14^ or morphometric examination.^15^

## CASE HISTORY

**Figure 1.**
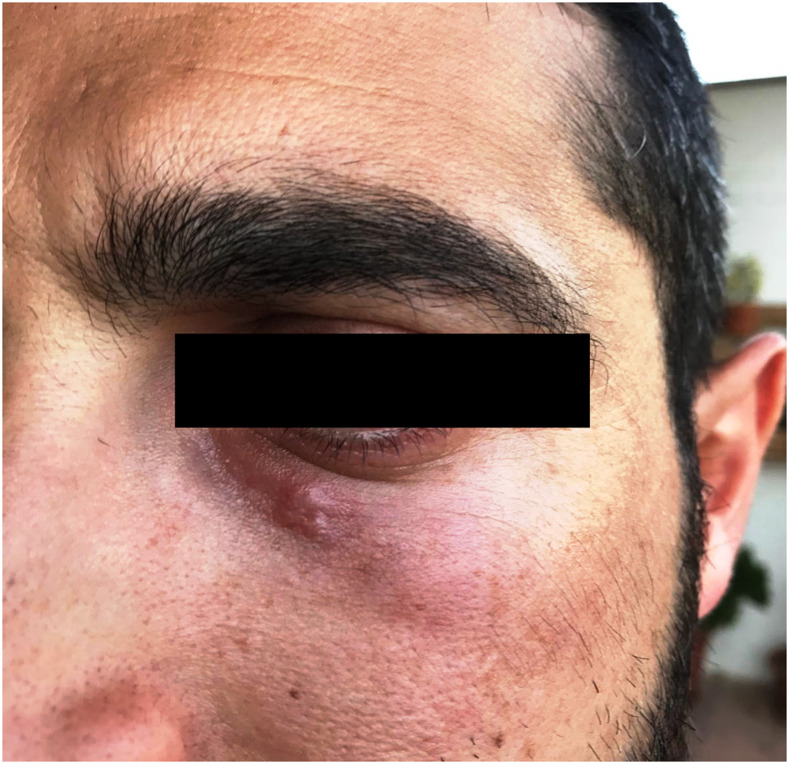
The nodule with subpalpebral edema of the patient.

In March 2019, a 35-year-old man, resident in Apulia, a region in the south of Italy, presented to a local hospital because of a subpalpebral edema surrounding a periocular nodule, about 1.5 cm^2^ large, localized under the left lower eyelid (Figure 1). The anamnestic record did not evidence relevant pathologic events, but the patient reported a two-week stay in Tanzania five months before the hospital admission. He also declared no further movements outside Apulia after having been returned to Italy.

The hematologic parameters were within the normality range, and the hemochromocytometric profile was regular (Table 1). A sample of serum underwent *Acanthocheilonema vitae* ELISA (Bordier Affinity Products, Crissier, Switzerland), a pan-filarial IgG-detecting immunoenzymatic assay,^16^ which returned a value of 0.46, thus below the positivity threshold, established at 1.

**Table 1:** Hematologic parameters of the patient

Hematologic parameter	Value	Normality range
Red blood cell count	5.10 × 10^12^/L	(4.50–5.90) × 10^12^/L
Hemoglobin	162 g/L	(135–170) g/L
Hematocrit	46.4%	(41.0–53.0)%
Mean cell volume	90.7 × 10^−15^ L	(80.0–99.0) × 10^−15^ L
Mean cell hemoglobin	31.5 × 10^−12^ g	(27.0–24.0) × 10^−12^ g
Mean cell hemoglobin concentration	349 g/L	(310–360) g/L
Red cell distribution width	12.9%	(11.0–15.0)%
White blood cell count	7.99 × 10^9^/L	(4.5–10.5) × 10^9^/L
Neutrophils	5.71 × 10^9^/L	(1.9–8.0) × 10^9^/L
	71.42%	(38.00–71.00)%
Eosinophils	0.08 × 10^9^/L	≤ 0.06 × 10^9^/L
	0.96%	≤ 6.00%
Basophils	0.04 × 10^9^/L	≤ 0.02 × 10^9^/L
	0.44%	≤ 1.50%
Lymphocytes	1.58 × 10^9^/L	(1.0–5.2) × 10^9^/L
	19.80%	20.00–50.00%
Monocytes	0.59 × 10^9^/L	(0.2–1.0) × 10^9^/L
	7.35%	4.00–10.00%
Platelet count	250 × 10^9^/L	(150–350) × 10^9^/L
Mean platelet volume	9.2 × 10^−15^ L	(7.00–12.50) × 10^9^/L
Platelet distribution width	16.7%	(10.0–65.0)%
Total IgE	2.21 × 10^5^ U/L	< 10.0 × 10^5^ U/L

**Figure 2.**
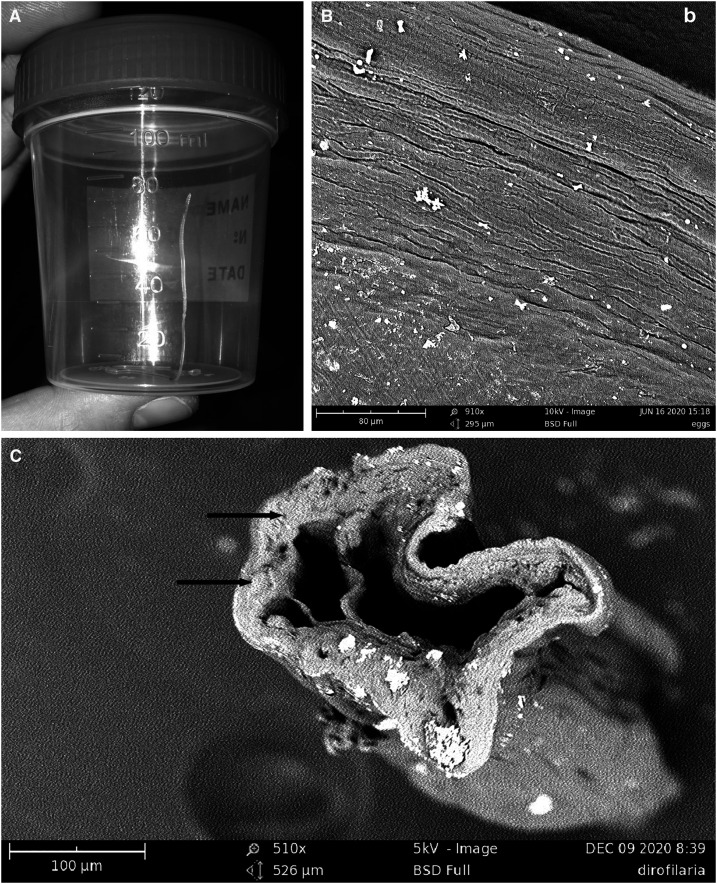
(**A**) Adult worm surgically collected from the nodule. (**B**) A particular of the SEM micrography evidencing the longitudinal ridges typical of *Dirofilaria repens*. (**C**) SEM micrography of a section of the adult worm excised from the patient. Lateral chords are indicated by arrows.

The nodule was surgically inspected, and two adult filarial parasites, 10 and 5 cm long, respectively, were removed (Figure 2A). The SEM analysis of the specimens revealed morphological features compatible with *D. repens*, including longitudinal ridges on the external cuticle, typical of *D. repens*,^17^ Candidatus *Dirofilaria hongkongensis*,^18^ and *Dirofilaria ursi*,^19^ and two large lateral chords, considered *D. repens* specific (Figure 2B).^17^

The Knott’s test resulted negative, but LAMP^12^ and PCR^17^ detected *D. repens* DNA from a specimen of blood EDTA. Total genomic DNA was also extracted and purified from a section of the removed worms and used as a template in the PCR^17^ to confirm the identification. The nucleotide sequences of the amplification products from blood (GenBank accession number MT683121) and worm (MT683122) were 100% identical between themselves, and one of them was aligned by ClustalO with a representative panel of corresponding sequences from *D. repens* present in GenBank. The maximum likelihood phylogeny was inferred by the mean of PhyML 3.0 by applying the generalized time-reversible model, selected by FindModel. The sequence of the isolate was part of a heterogeneous cluster (Supplemental Figure 1) that included sequences from *D. repens* isolated in Germany, Italy, and Croatia, 100% identical among themselves. Conversely, the sequence from this study was slightly distant (*p*-distance = 0.0015) from them.

After surgical removal of the adult worms, the patient was treated with doxycycline, and no resurgence was reported after one year.

## DISCUSSION

The diagnosis of human dirofilariasis mostly relies on the morphologic identification of the worm after biopsy or surgical removal.^2^ Preventive analyses such as ultrasound examinations or pan-filarial IgG tests might be useful to better address surgery, but they cannot provide a definite diagnosis before the morphometric inspection. The currently available ELISA test may be relatively beneficial, but it lacks specificity as it may detect IgG produced after infestation by several species, such as *Brugia malayi*, *Loa loa*, *Mansonella* sp., *Onchocerca volvulus*, and *Wuchereria bancrofti*, other than *Dirofilaria immitis* and *D. repens*.^16^ On the other side, morphologic identification may be time- and labor-consuming, and it requires operators with specific expertise, not always available in all diagnostic laboratories. Nevertheless, the here reported case also poses a matter of sensitivity. Molecular assays managed to detect and identify *D. repens* DNA from the patient’s blood, but no microfilariae, or even L3 larvae, were observed by the Knott’s test, as expected because of the infrequency of microfilaremia in humans.^13^ In addition, the case evidenced the absence of potential hematological markers for dirofilariasis because no parameter was significantly out of range. The absence of eosinophilia and the low level of IgE, along with the negative results gathered by the IgG-detecting ELISA, reflect the poor efficacy of hematologic or serological diagnostic tools, already known.^20^ Conversely, the case highlights the possible interest of molecular tools due to their sensitivity and specificity, already proved in vitro and, less frequently, infield. It is tempting to speculate that, in humans, the circulation of larval stages of *D. repens* in the patient’s blood occurs at very low concentration, insomuch that it can be detectable only by highly sensitive methods, such as the molecular ones. Further studies might be aimed to clearly ascertain which stages are vesiculated by the bloodstream and their concentration.

The value of molecular tests is also enhanced by its potential contribution to the differential diagnosis between loiasis and dirofilariasis, especially for those patients declaring stays in tropical regions considered endemic for *L. loa*. The latter infestation has usually worse outcomes than dirofilariasis, but it is often (but not always) associated with eosinophilia.^21^ In fact, despite uncommon, periocular localization of *L. loa* has been reported.^22^

Finally, the present report adds further considerations about the possibility to import cases of dirofilariasis from Africa. The infestation has already been diagnosed in patients returning from Malaysia and Botswana to Spain^23^ or from Senegal to France^24^ and Belgium (the latter case with the evidence of microfilaremia),^25^ but no clear pieces of evidence were provided about the acquisition of the infection in foreign countries. Unfortunately, to date, no sequences of *D. repens* collected from Central and Southern Africa are available for comparison, despite it being isolated or detected from dogs in Tanzania^26^ and Cape Verde.^27^ Despite the scarce clonality of *D. repens* population, they could offer very useful information about the worldwide circulation of the parasite, and a potential starting point to assess the international transmission routes, which could be directed to, or even originating from, Europe.

## Supplemental figure

Supplemental materials
