# Supplementary figures and images for: Case Report: Molecular Detection of Dirofilaria repens in an Italian Patient after a Stay in Tanzania
Source: Am J Trop Med Hyg. 2021 May 3;104(6):2042–5. doi: 10.4269/ajtmh.20-1360 (PMC8176467; doi:10.4269/ajtmh.20-1360)

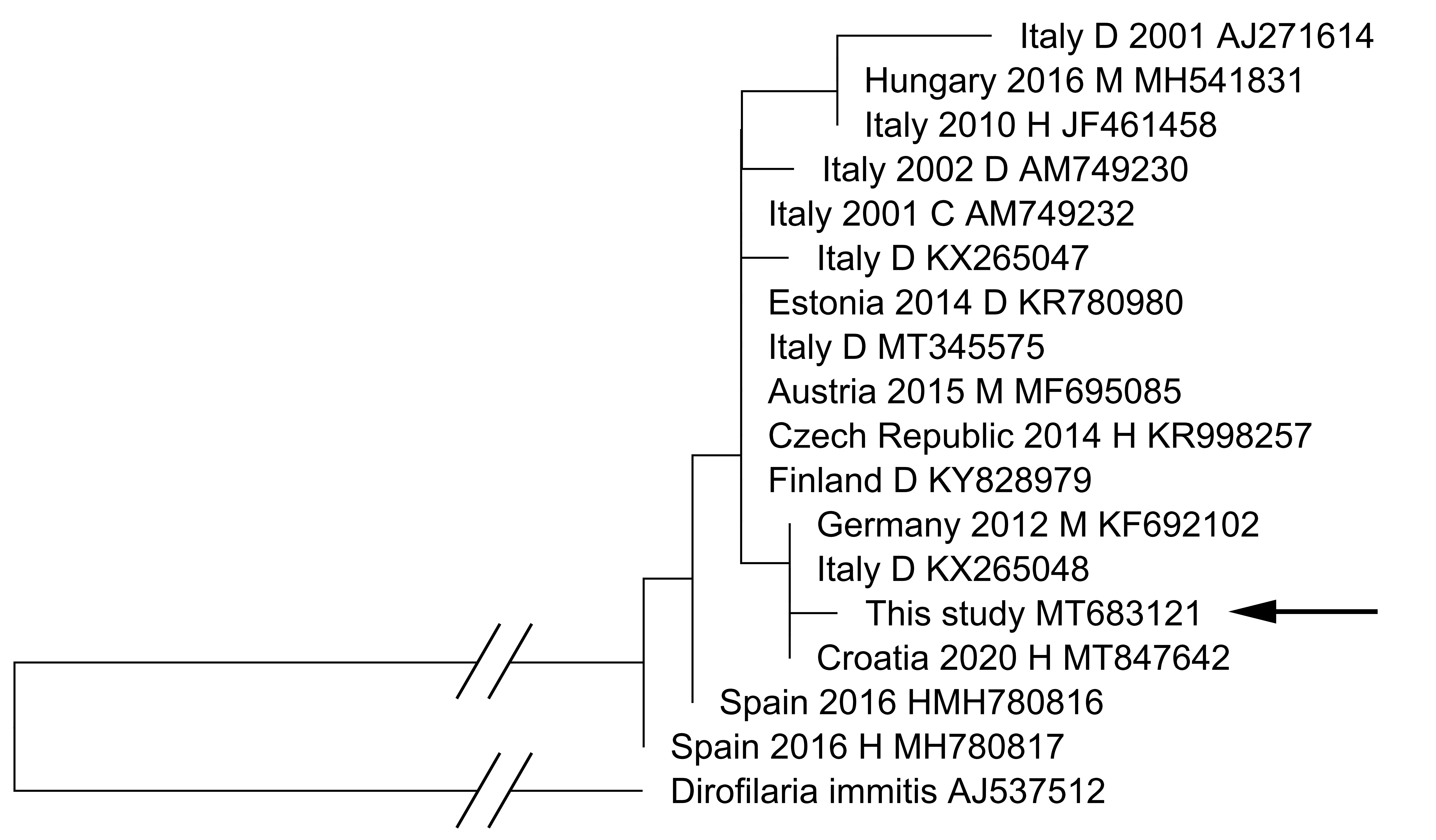

Supplement: Supplementary file 2 [file tpmd201360.SF1.tif]
